# Exposure to intrauterine inflammation alters metabolomic profiles in the amniotic fluid, fetal and neonatal brain in the mouse

**DOI:** 10.1371/journal.pone.0186656

**Published:** 2017-10-19

**Authors:** Amy G. Brown, Natalia M. Tulina, Guillermo O. Barila, Michael S. Hester, Michal A. Elovitz

**Affiliations:** Maternal Child Health Research Center, Department of Obstetrics and Gynecology, Perelman School of Medicine, University of Pennsylvania, Philadelphia, Pennsylvania, United States of America; Johns Hopkins University, UNITED STATES

## Abstract

**Introduction:**

Exposure to prenatal inflammation is associated with diverse adverse neurobehavioral outcomes in exposed offspring. The mechanism by which inflammation negatively impacts the developing brain is poorly understood. Metabolomic profiling provides an opportunity to identify specific metabolites, and novel pathways, which may reveal mechanisms by which exposure to intrauterine inflammation promotes fetal and neonatal brain injury. Therefore, we investigated whether exposure to intrauterine inflammation altered the metabolome of the amniotic fluid, fetal and neonatal brain. Additionally, we explored whether changes in the metabolomic profile from exposure to prenatal inflammation occurs in a sex-specific manner in the neonatal brain.

**Methods:**

CD-1, timed pregnant mice received an intrauterine injection of lipopolysaccharide (50 μg/dam) or saline on embryonic day 15. Six and 48 hours later mice were sacrificed and amniotic fluid, and fetal brains were collected (n = 8/group). Postnatal brains were collected on day of life 1 (n = 6/group/sex). Global biochemical profiles were determined using ultra performance liquid chromatography/tandem mass spectrometry (Metabolon Inc.). Statistical analyses were performed by comparing samples from lipopolysaccharide and saline treated animals at each time point. For the P1 brains, analyses were stratified by sex.

**Results/Conclusions:**

Exposure to intrauterine inflammation induced unique, temporally regulated changes in the metabolic profiles of amniotic fluid, fetal brain and postnatal brain. Six hours after exposure to intrauterine inflammation, the amniotic fluid and the fetal brain metabolomes were dramatically altered with significant enhancements of amino acid and purine metabolites. The amniotic fluid had enhanced levels of several members of the (hypo) xanthine pathway and this compound was validated as a potential biomarker. By 48 hours, the number of altered biochemicals in both the fetal brain and the amniotic fluid had declined, yet unique profiles existed. Neonatal pups exposed to intrauterine inflammation have significant alterations in their lipid metabolites, in particular, fatty acids. These sex-specific metabolic changes within the newborn brain offer an explanation regarding the sexual dimorphism of certain psychiatric and neurobehavioral disorders associated with exposure to prenatal inflammation.

## Introduction

Exposure to intrauterine inflammation has been demonstrated to induce fetal brain injury and is associated with adverse neurobehavioral disorders in offspring [1–5]. Specifically, maternal bacterial and viral infections during pregnancy increase the risk of developing neuropsychiatric disorders such as schizophrenia, autism spectrum disorder (ASD) and cognitive delay [6–10]. Several of these psychiatric disorders show differential prevalence between males and females. Schizophrenia and ASD have increased incidence in males [11], suggesting that the sex of the fetus may play an important role in determining the physiological response to inflammation and the subsequent development of these syndromes.

Sex differences in the brain are apparent during perinatal development. These differences are the result of a combination of gonadal steroid influences as well as a chromosomal contribution. There is an undeniable sex bias in most if not all neuropsychiatric and neurological disorders [12]. In fact, being male imparts risk for the development of ADHD and Tourette’s Syndrome whereas being female confers a level of protection against the development of these disorders [11]. It is clear that sex programs the fetal brain and has lasting behavioral and psychological impacts. It is only by interrogating the sexual differences in brain development that we can increase our understanding of the sexual dimorphism of neurological and psychiatric illnesses.

Animal models representing systemic maternal infection or local intrauterine inflammation have been critical in furthering our understanding of inflammation-induced fetal brain injury. We and others have shown that exposure to prenatal inflammation results in significant injury to the fetal brain including loss of pro-oligodendrocytes, a significant alteration in neuronal development, post-natal changes in gene expression as well as altered behavior [1–4,13–15]. Others have shown that systemic inflammatory stimuli such as viral infections or simply prenatal exposure to the viral mimetic poly I:C causes altered brain structure, neurochemical changes and behavioral deficits in offspring [5,16–19]. Despite this body of work demonstrating an association between prenatal inflammation and adverse neurological outcomes, the mechanism by which prenatal inflammation negatively impacts the developing brain is not well defined. Furthermore, there are no reliable biomarkers or predictors of fetal brain injury. Therefore, we performed metabolomics, a novel, discovery based approach, to further investigate the underlying mechanisms of inflammation-induced fetal and neonatal brain injury.

*Metabolomics* is the large-scale study of small molecules, commonly known as metabolites, within cells, bio fluids, tissues or organisms [20]. Most recently, metabolomic profiles have been deemed useful in differentiating health versus disease states in a variety of syndromes resulting in more than 1000 publications. Recently, investigators have been using metabolomics to profile serum or plasma in search of biomarkers and to explore the mechanisms of inflammatory, hypoxia/ischemia and traumatic brain injuries [21–23]. Specifically, Keller *et al*. and Chun *et al*. sampled their animal models of brain injury temporally which provided snapshots of the metabolome hours to days after the injury. We know from our model of inflammation-induced fetal brain injury that the neuronal injury is persistent and is evident 48 hours after the insult, long after the acute inflammatory response [3]. Additionally, these researchers identified compounds which could not only act as biomarkers of brain injury, but also indicate metabolic pathways that could be critical for the pathogenesis of neuronal and glial damage.

Despite the vast amount of research exploring the metabolome in other conditions, there has been no comprehensive study of the amniotic fluid and the brain metabolome post-exposure to prenatal inflammation. Our overarching hypothesis is that exposure to intrauterine inflammation begets metabolic changes in the fetus and fetal brain that may be mechanistically involved in initiating and perpetuating fetal and neonatal brain injury. For these studies, we sought to assess 1) if exposure to intrauterine inflammation alters the metabolomic profile of the amniotic fluid and fetal brain after acute exposure to inflammation, 2) if metabolic profiles remained disrupted beyond the acute exposure and 3) if metabolic changes would be evident in the postnatal brain of exposed pups and whether there would be a sex-specific effect.

## Materials and methods

### Animal model

**Fig 1:**
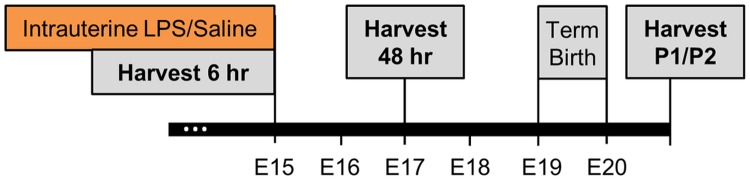
CD-1 timed-pregnant females were given an intrauterine injection (IUI) of either LPS or saline at gestational day 15. Tissues were harvested at three time points: 6 hours (n = 8 per group, total n = 16), 48 hours (n = 8 per group, total n = 16) and post-natal day (P) 1. About 50–60% of the dams delivered preterm within the first 24 hours. Some of the dams were used to collect samples at 48 hours after administration. The rest of the dams delivered at term. At P1, pups were euthanized and tissues harvested discriminated by gender and experimental condition (n = 6 per group/treatment, total n = 24).

A previously described mouse model of intrauterine inflammation, which results in fetal and postnatal brain injury [1,3,4,24], was utilized for these studies. For all experiments, CD-1, timed pregnant mice were purchased from Charles River Laboratories (Wilmington, MA). Animals were shipped 8–12 days after mating and allowed to acclimate in our facility for 3–7 days. Briefly, a mini-laparotomy was performed under isoflurane anesthesia on CD-1, timed pregnant mice (Charles River Laboratories, Wilmington, MA) at gestational day 15 (E15), with normal gestation being 19–20 days. The right lower uterus was exposed allowing visualization of the lower two gestational sacs. Mice then received intrauterine injections of liposaccharide (LPS) from *Escherichia coli* (055:B5, Sigma, St Louis, MO, L2880, 50ug/100μl phosphate buffered saline/animal; LPS-treated group), or PBS (100μl/animal; control, saline-treated group). Surgical incisions were closed using staples and dams were allowed to recover for 6 and 48hrs prior to tissue collection (n = 8/group per time period). A separate group of LPS and saline injected animals was allowed to deliver and their offspring was separated by sex and sacrificed on postnatal day 1 (n = 6/group/gender). A male and female pup was collected from each dam to represent the litter (n = 6 male/controls, n = 6 male/LPS, n = 6 female/controls, n = 6 female/LPS). The experimental design is contained within Fig 1. All experiments were performed in accordance with the National Institute of Health Guidelines on Laboratory Animals with approval from the University of Pennsylvania’s Animal Care and Use Committee (protocol number 804658).

### Tissue collection

Six and forty eight hours post-injection, all dams from the different treatment groups were euthanized (n = 8/group) and fetal tissues, including brain and amniotic fluid, were collected. Specifically, for the brain dissection, the craniums were removed and fetal brains were collected and pooled from fetuses within the four gestational sacs proximal to the injection site (4 fetal brains, from 1 dam = n = 1). Amniotic fluid was extracted from gestational sacs using 1ml syringes with 18 gauge needles, centrifuged at 3,000g for 5min. and the supernatants were flash frozen in liquid nitrogen. For the postnatal experiments, the offspring was separated by sex using physical examination and euthanized on P1 (n = 6/group/gender). Neonatal whole brains were isolated after separating meninges and processed for tissue collection. Tissues were flash frozen immediately after collection and stored at -80°C for future processing.

### Metabolic profiling of fetal brain and amniotic fluid

#### Sample preparation

The analysis of the metabolic content was performed by (Metabolon, Inc., Research Triangle Park, NC, USA). To ensure effective retrieval of diverse metabolic compounds, tissue samples were deproteinized by methanol precipitation under vigorous shaking for 2min. (Glen Mills GenoGrinder 2000) followed by centrifugation. Recovery standards were added to all samples before extraction for quality control purposes.

#### Ultra-performance liquid chromatography—Tandem mass spectrometry (UPLC-MS/MS)

Obtained extracts were split into equal portions, dried and reconstituted in solvents suitable for four different UPLC-MS/MS methods: 1) reverse phase (RP) UPLC-MS/MS with positive ion conditions, optimized for hydrophilic compounds; 2) (RP) UPLC-MS/MS with positive ion conditions, optimized for hydrophobic compounds; 3) (RP) UPLC-MS/MS method with negative ion conditions, and 4) hydrophilic interaction liquid chromatography (HILIC)/ UPLC-MS/MS with negative ion conditions. A Waters ACQUITY UPLC system supplied with either C18 (Waters UPLC BEH C18-2.1x100mm, 1.7μm) or HILIC (Waters UPLC BEH Amide 2.1x150mm, 1.7μm) columns was utilized. The extracts were eluted with: water and methanol, both containing 0.05% per fluoropentanoic acid (PFPA) and 0.1% formic acid (FA) (method 1); methanol, acetonitrile and water with 0.05% PFPA and 0.01% FA (method 2); water and methanol with 6.5mM ammonium bicarbonate (pH8.0) (method 3), and water and acetonitrile containing 10mM ammonium formate (pH 10.8) (method 4). Mass spectrometry was performed using a Thermo Scientific Q-Exactive high resolution/accurate mass spectrometer interfaced with a heated electrospray ionization source and Orbitrap mass analyzer operated at 35,000 mass resolution. The scans alternated between MS and data-dependent MS^n^ scans and covered the range 70–1000 m/z.

#### Quality control

The following samples were included in the analysis to optimize the method: a technical replicate made either of a human plasma extract or combined aliquots of all experimental samples and blank samples containing water and organic solvents. In addition, quality control standards mixed with the samples of interest were used for evaluating instrument performance.

#### Data analysis and normalization

All samples were accessioned into the Metabolon Laboratory Information Management System (LIMS) and assigned a unique identifier. Data analysis was accomplished using the data extraction and peak identification software, the data processing tools for quality control and compound identification, and a collection of information interpretation and visualization tools. Individual metabolites were identified by comparison to the library of over 3300 known, commercially available chemical standards, based on retention time/index (RI), mass/charge (mz) and chromatographic data. The measurements for each compound were acquired multiple times and normalized to volume (amniotic fluid samples) or protein (brain tissue).

#### Random forest

Random forest (RF) is a supervised classification technique reporting on the consensus of a large number of decision trees. In this study the RF plots utilized 30 biochemicals to discriminate between the LPS-exposed and saline-exposed tissues/fluid. An accuracy of 50% is expected by random chance. Any accuracy greater than 50% is better than random chance.

### Enzymatic assay for measuring concentrations of xanthine and hypoxanthine

To determine the levels of xanthine and hypoxanthine in fetal brain an assay kit that measures the enzymatic activity of xanthine oxidase was utilized according to manufacturer’s instructions (Abcam, ab155900, Cambridge, UK). For protein extraction, 10mg of fetal brain tissue was homogenized in 100μl of ice cold assay buffer and the obtained homogenates were diluted 3:5. The amount of xanthine and hypoxanthine was determined based on absorption measured at 570nm using SpectraMax M2/M2^e^ (Molecular Devices) and SoftMaxPro 5.2 software.

### Statistical analyses

All statistically significant values are expressed as fold-change and calculated as a ratio of LPS-treated versus control (saline exposed). Statistical analyses of the metabolomic profiling in amniotic fluid, and fetal brains were performed using Welch’s two-sample *t*-test. For the post-natal studies a two-way analysis of variance (ANOVA) was performed. Only the metabolites which produced p-values<0.05 were considered significantly different between the LPS- and saline-treated groups. For the gender and treatment effects the df is 1, interaction = 1, and error = 23. Specifically, for the neonatal brains, a group/gender interaction was investigated and there were 9 compounds that were significantly altered in a sex-specific manner (P < 0.05). Therefore for the post-natal study, the metabolic profiles of the neonates were further stratified by the sex of the neonate. The data, obtained using the enzymatic xanthine/hypoxanthine assay, were analyzed using Welch’s two-sample *t*-test.

## Results

### Metabolic response in the amniotic fluid 6 hours after intrauterine inflammation

**Fig 2:**
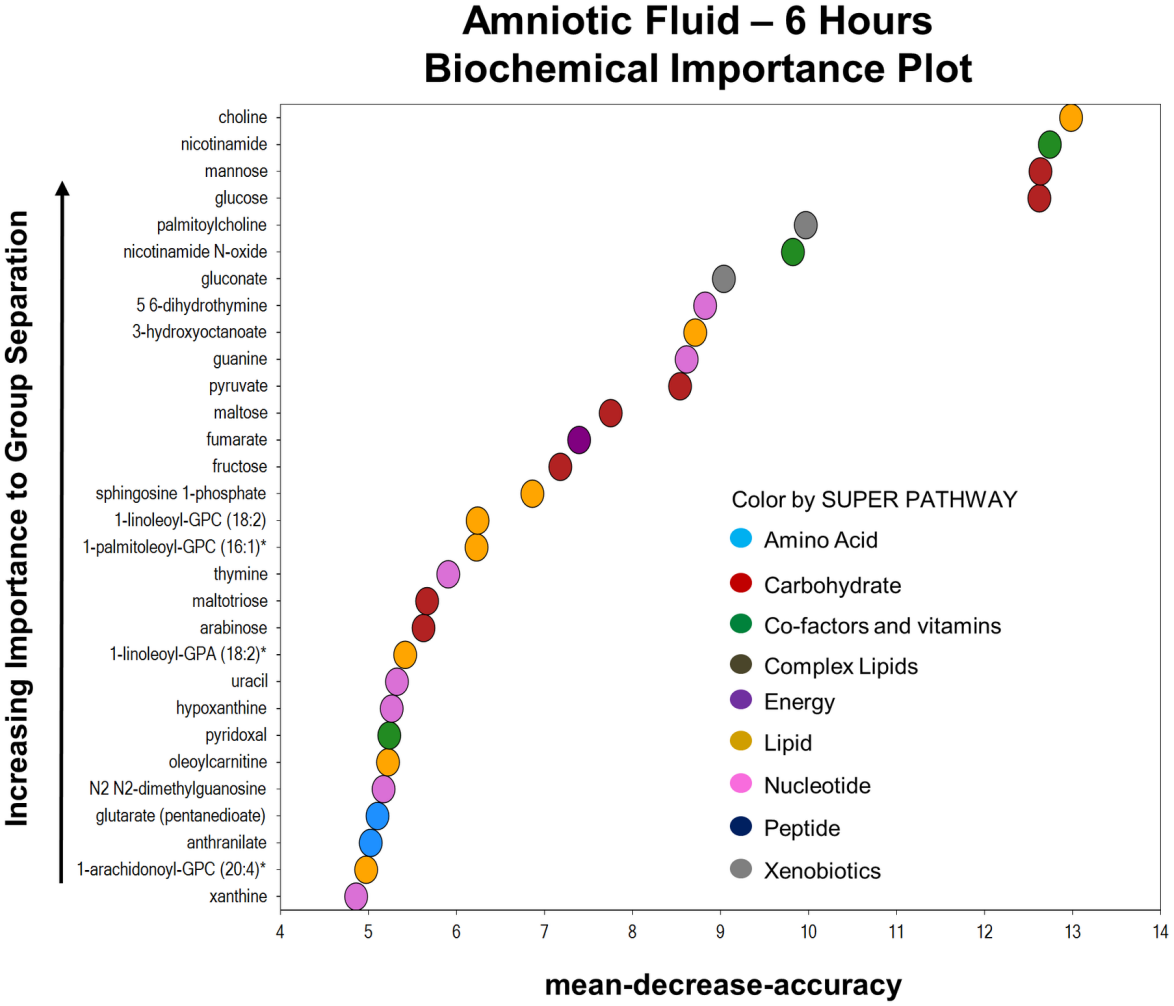
The metabolic profile of the amniotic fluid 6 hours after exposure to inflammation. Random forest plot of top 30 biochemicals present in the amniotic fluid based on their importance in separating the metabolic profiles of the saline and LPS-exposed groups. The predictive accuracy of the plot was 87%.

At 6 hours post-exposure to *in utero* inflammation, there was a profound change in the metabolome of the amniotic fluid. Sixty out of 444 total biochemicals were elevated and 21 metabolites had reduced levels. The random forest plot ranks the top 30 biochemicals that can be used to discriminate between the LPS-exposed versus saline-exposed fetuses. The biochemicals at 6 hours belong to every super pathway family: amino acid, carbohydrate, co-factors and vitamins, complex lipids, energy, lipids, nucleotides, peptides and xenobiotics. The predictive accuracy of the random forest plot was 87%. (Fig 2)

#### Amino acid metabolism

Twenty amino acid metabolites out of 136 total biochemicals detected in this category were significantly enhanced, while only one showed a decline. Components of the glutamate, histidine, lysine, tryptophan, and branched chain amino acid metabolic pathways were increased in LPS-exposed amniotic fluid compared to controls. While most metabolites showed less than 3-fold differences between LPS- and saline-treated groups, GABA, glutarate and kynurate had a greater than 3-fold enhancement (Table 1).

**Table 1:** Amino acid metabolites altered in amniotic fluid, 6 hours after exposure to prenatal inflammation.

Amino Acid Metabolism	Biochemical	Fold Change *
Glutamate Metabolism	GABA	3.51
Histidine Metabolism	N-acteylhistidine	1.41
	3-methylhistidine	1.46
Lysine Metabolism	N2-acteyl lysine	2.01
	Glutarate	3.38
	N2,N6 Diacetyllysine	1.34
Tryptophan Metabolism	Kynurate	3.03
	Anthranilate	2.01
	Indole-3-carboxylic acid	1.43
Branched Chain Amino Acids	N-acetylleucine	2.94
	4 methyl-2-oxopentanoate	1.25
	Beta-hydroxyisovalerolycarnitine	1.36
	Alpha-hydroxyisovalerate	1.53
	Methylsuccinate	1.31
	Isoleucine	1.32
	N-acetylisoleucine	0.84
	2-methylbutyrylcarnitine	1.41

Asterisk indicated statistical significance with a p-value < 0.05. Fold changes greater than 1 indicate a statistically significant (p < 0.05) increase in abundance and fold changes less than one indicate a statistically significant (p < 0.05) decrease in metabolite abundance.

#### Nucleotide metabolism

**Fig 3:**
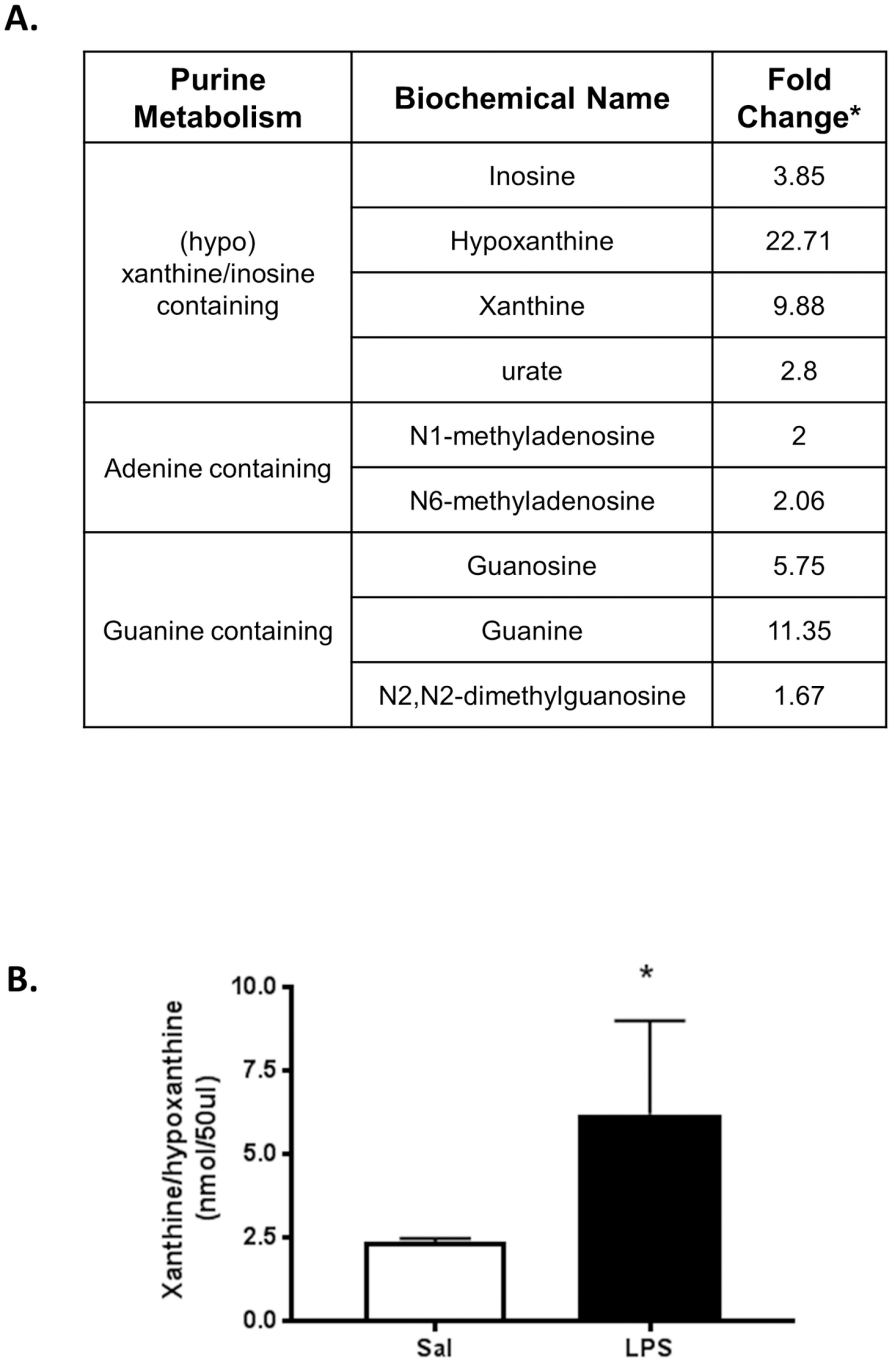
Levels of purine metabolites in amniotic fluid, 6 hours after exposure to inflammation. A. Table displaying increased purine metabolite abundance. B. Validation of xanthine/hypoxanthine in amniotic fluid samples utilizing a commercially available assay. Asterisk indicates p < 0.05.

Multiple purine metabolic products were dramatically increased. Specifically, (hypo) xanthine/inosine containing compounds such as inosine, hypoxanthine, xanthine, and urate were enhanced 3.85, 22.71, 9.88 and 2.80-fold, respectively. Adenine and guanine containing purine metabolites were enhanced as well in the inflammation-exposed amniotic fluid compared to controls (Fig 3A). This upregulation was verified via a commercially available assay, which measured the combined content of xanthine and hypoxanthine. Obtained results confirmed the multi-fold increase in the content of these metabolites in the amniotic fluid as shown earlier using UPLC-MS/MS (Fig 3B).

### Metabolic response in the amniotic fluid 48 hours after intrauterine inflammation

**Fig 4:**
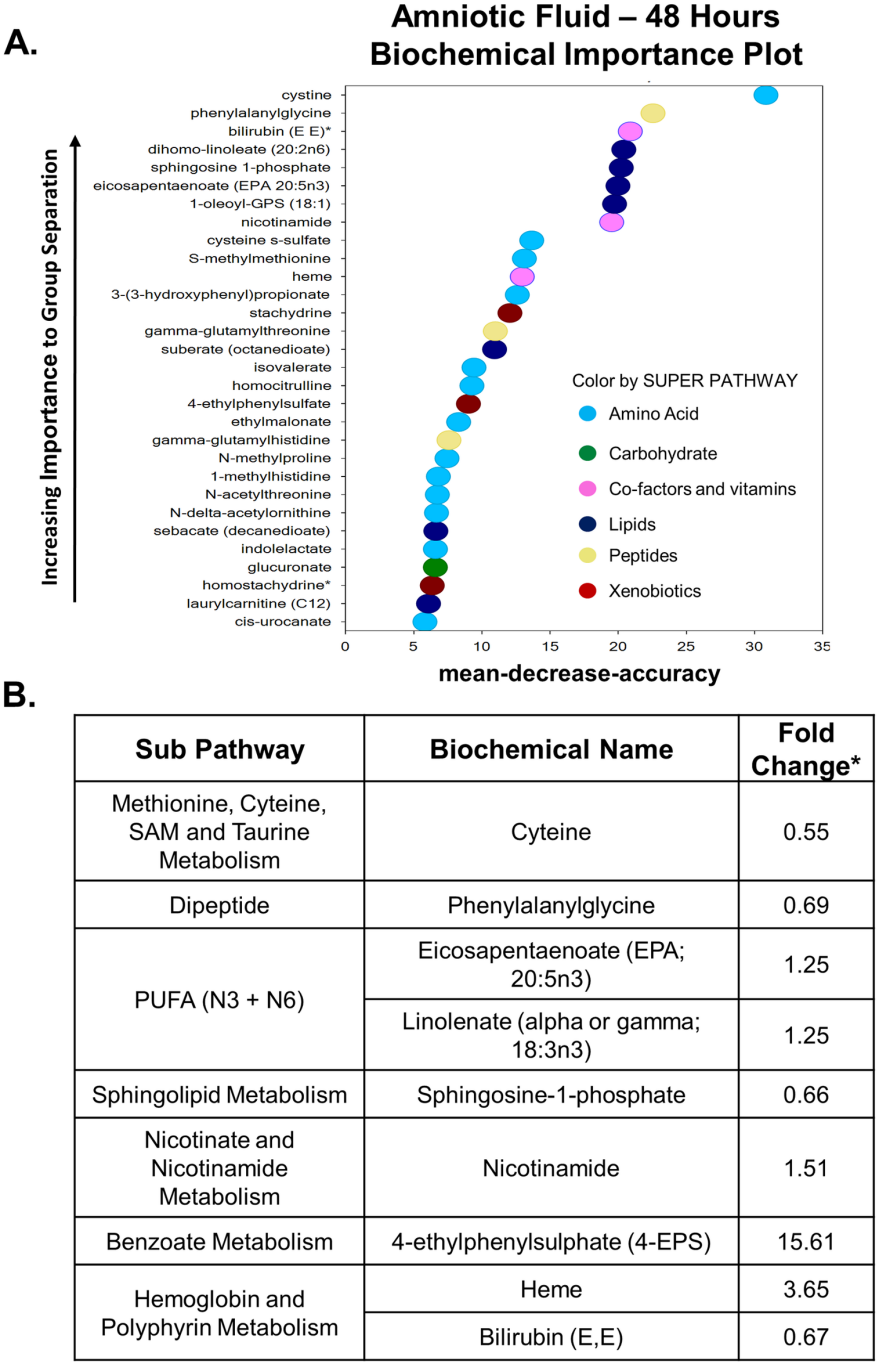
The metabolic profile of the amniotic fluid 48 hours after exposure to prenatal inflammation. A. Random forest plot of top 30 biochemicals present in the amniotic fluid, 48 hours after exposure to inflammation, based on their importance in separating the metabolic profiles of the saline and LPS exposed groups. The predictive accuracy was 56%. B. Nine total metabolites were altered in the amniotic fluid at this time point. Asterisks indicate statistical significance with a p-value < 0.05.

In contrast to the global metabolic changes in the amniotic fluid observed 6 hours after intrauterine inflammation, at the 48 hour time point, the metabolic profile was less dramatically altered. Specifically, within the amniotic fluid, only 9 out of 500 total metabolites were different between LPS and saline-treated groups. Five metabolites were significantly upregulated and 4 were significantly decreased. The predictive accuracy of the random forest plot was 56% (Fig 4A). Fig 4B contains the nine biochemicals that were altered in the amniotic fluid 48 hours after LPS-exposure. Compared to less than 2-fold changes in other metabolite levels, 4-ethylphenyl sulfate was dramatically enhanced in the amniotic fluid of LPS-exposed fetuses compared to controls (15-fold, p < 0.05).

### Metabolic response in the fetal brain 6 hours after intrauterine inflammation

**Fig 5:**
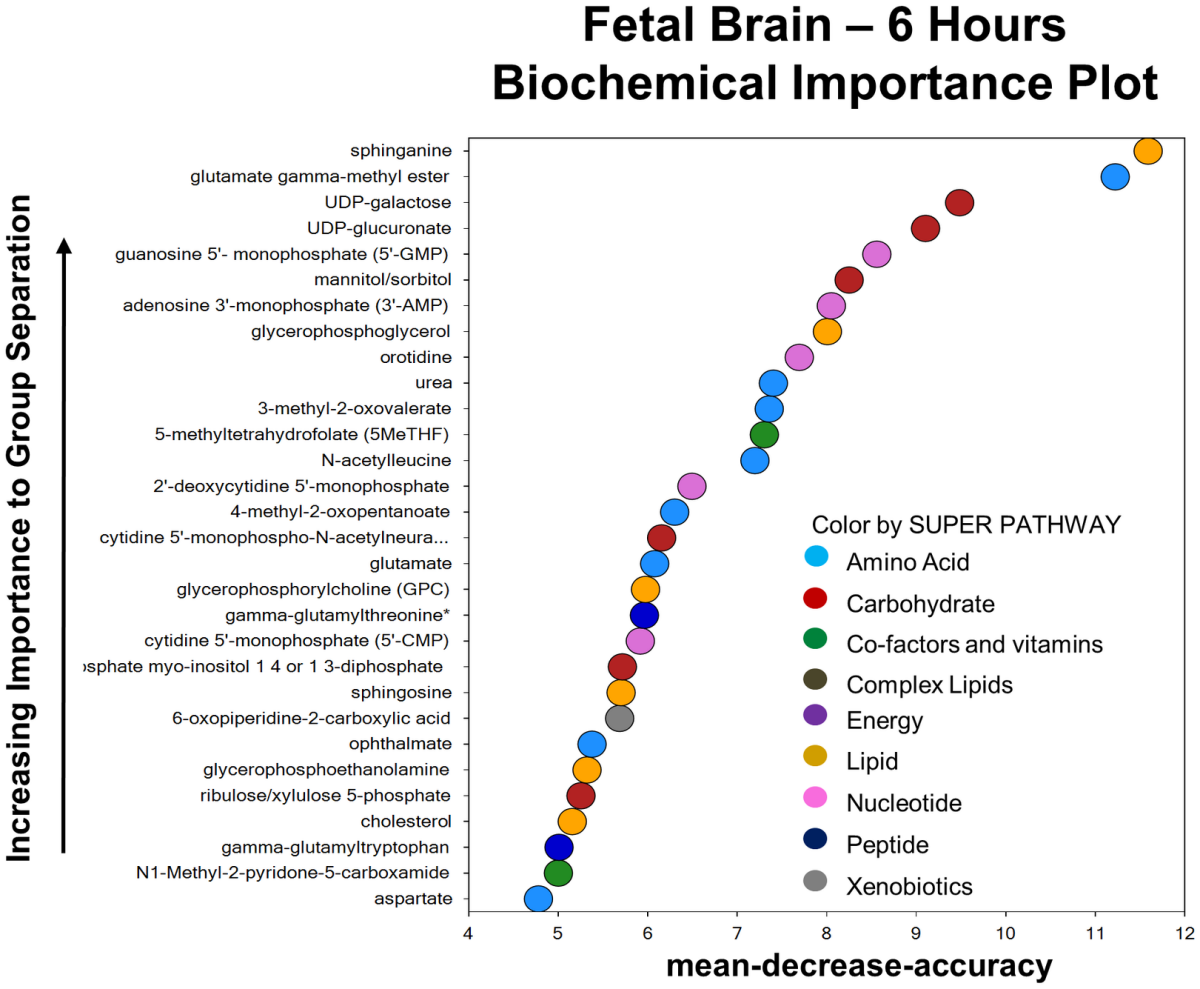
The metabolic profile of the fetal brain six hours after exposure to inflammation. Random forest plot of top 30 biochemicals present in the fetal brain 6 hours post exposure based on their importance in separating the metabolic profiles of the saline and LPS exposed groups. The predictive accuracy was 75%.

At 6 hours post-exposure to *in utero* inflammation, there was a profound change in the metabolome of the fetal brain. Sixty-five out of 467 total biochemicals were elevated and 50 metabolites had reduced levels (P < 0.05). The 30 biochemicals used to construct the random forest plot belonged to every super pathway family: amino acid, carbohydrate, co-factors and vitamins, complex lipids, energy, lipids, nucleotides, peptides and xenobiotics. The predictive accuracy of the random forest plot was 75%. (Fig 5)

#### Amino acid metabolism

Out of the 157 total amino acids detected, 30 were significantly enhanced and 12 were diminished in the LPS-exposed fetal brain compared to their saline exposed counterparts. Biochemicals of the following amino acid families were altered: glutamate, lysine, tryptophan, alanine, aspartate, polyamine, histidine and branched chain amino acid metabolism were significantly altered (Table 2). Most compounds changed less than two-fold except for branched chain amino acids 4-methyl-2-oxopentanoate and 3-methyl-2-oxovalerate, which increased 9.24 and 8.04-fold, respectively.

**Table 2:** Alterations in amino acids in the fetal brain six hours post-LPS exposure.

Amino Acid Metabolism	Biochemical Name	Fold Change*
Glutamate Metabolism	GABA	1.25
Lysine Metabolism	Glutarate	1.33
Tryptophan Metabolism	Kynurenate	2.20
	Tryptophan	1.47
Alanine and Aspartate Metabolism	Aspartate	0.55
Polyamine Metabolism	Putrescine	1.12
Histidine Metabolism	Histidine	1.44
Branched Chain Amino Acid Metabolism	Leucine	1.44
	N-acteylleucine	2.06
	4-methyl-2-oxopentanoate	9.42
	Beta-hydroxyisovaleroylcarnitine	0.87
	Alpha-hydroxyisovalerate	1.93
	Isoleucine	1.44
	N-acetylisoleucine	1.46
	3-methyl-2-oxovalerate	8.04
	2-methylbutyrylcarnitine (C5)	1.74
	2-hydroxy-3-methylvalerate	2.47
	Ethylmalonate	1.17
	Valine	1.55
	3-hydroxyisobutyrate	0.78
	Alpha-hydroxyisocaproate	2.94

Asterisk indicated statistical significance with a p-value < 0.05. Fold changes greater than 1 indicate an increase in abundance and fold changes less than one indicate a decrease in metabolite abundance.

#### Purine metabolism

The (Hypo) Xanthine/Inosine containing metabolites were significantly elevated in the fetal brain, 6 hours after exposure to intrauterine inflammation. Specifically, the following members of this pathway were all significantly elevated: hypoxanthine (1.73 fold), xanthine (2.42 fold), xanthosine (8.88 fold) and urate (2.34) (S1 Table).

### Metabolic response in the fetal brain 48 hours after intrauterine inflammation

**Fig 6:**
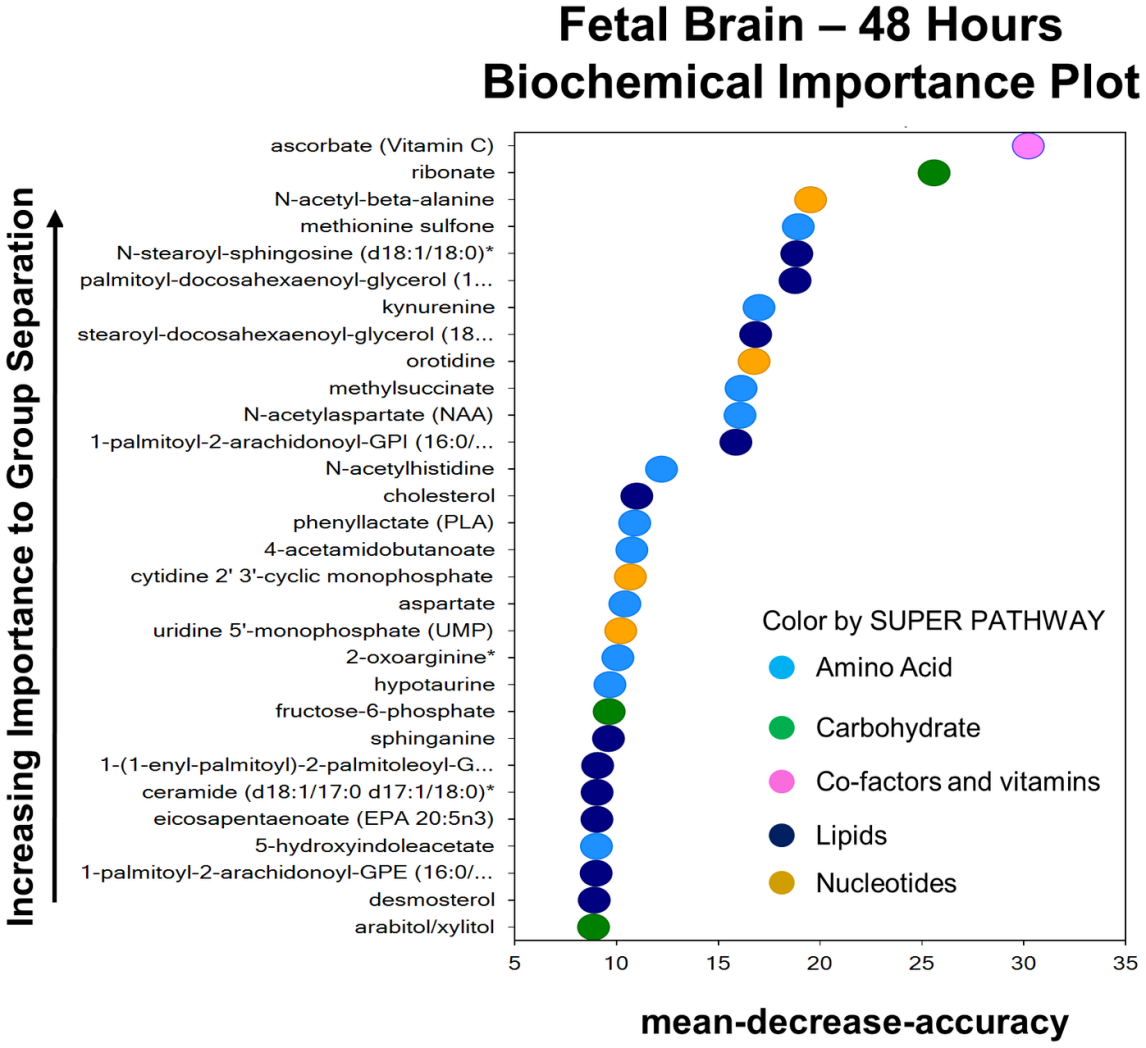
The metabolic profile of the fetal brain 48 hours after exposure to inflammation. Random forest plot of top 30 biochemicals present in the fetal brain at 48 hours post exposure based on their importance in separating the metabolic profiles of the saline and LPS exposed groups. The predictive accuracy was 69%.

Forty eight hours after exposure to inflammation, the metabolic fingerprint in the fetal brain is altered albeit in a less profound manner compared to the 6 hour time-point (Fig 6). Specifically, within the fetal brain, only 31 out of 512 metabolites were different between LPS and saline-treated groups at this time-point. Eleven compounds were elevated and 20 compounds were decreased in abundance in response to prenatal exposure to inflammation. The predictive accuracy of the random forest plot was 69% (Fig 6).

The majority of the metabolites used to drive the formation of the random forest plot are members of the amino acid and lipids families. Specifically, 10 out of 220 metabolites, mainly from phospholipid, monoacylglycerol, sphingolipid and sterol families; showed reduced levels compared to controls. Desmosterol and cholesterol, also downregulated 6 hours after intrauterine inflammation, remained decreased at this later time point, compared to controls (Table 3 and S1 Table).

**Table 3:** Alterations in lipid metabolites in the fetal brain 48 hours after prenatal exposure to inflammation.

Lipid Metabolism	Biochemical Name	Fold Change*
Phospholipid metabolism	1-palmitoyl-2-arachidonoyl-GPI (16:0/22:6)	0.86
	1-palmitoyl-2-oleoyl-GPG (16:0/18:1)	0.86
	1-palmitoyl-2-arachidonoyl-GPE (16:0/20:4)	0.89
	1-oleoyl-2-arachidonoyl-GPI (18:0/20:4)	0.88
Monoacylglycerol	Palmitoyl-docosahexaenoyl-glycerol (16:0/22:6)	0.80
	Stearoyl-docosahexaenoyl-glycerol (18:0/22:6)	0.80
Sphingolipid metabolism	Sphinganine	0.85
	N-stearolyl-sphingosine (d18:1/18:0)	0.83
Sterol	Desmosterol	0.86
	Cholesterol	0.87

Asterisk indicated statistical significance with a p-value < 0.05. Fold changes greater than 1 indicate an increase in abundance and fold changes less than one indicate a decrease in metabolite abundance.

### Changes in metabolic profile persist in the postnatal period and demonstrate sex specific differences

Sex-specific differences were detected in the brain metabolome of the control animals. Twenty-five compounds were altered when using sex as an independent variable (Table 4). Most compounds demonstrated less than a 2-fold alteration in abundance, except for N-steroyltaurine which was increased 3.22-fold in the female brain compared to their control male counterparts.

**Table 4:** Sex differences in the metabolic content of the neonatal brain in control animals.

Group	Biochemical Name	Fold Change (Female/Male)*
Amino Acids	1-methylhistamine	1.21
	S-adenosylhomocysteine (SAH)	0.82
	taurine	0.95
	gamma-glutamylvaline	1.25
Carbohydrates	lactate	0.81
	UDP-glucose	0.89
	guanosine-5'-diphosphofucose	0.84
	malate	0.84
Lipids	adrenoylcarnitine (C22:4)	1.83
	dihomo-linoylcarnitine (20:3n3 or 6)	1.56
	N-stearoyltaurine	3.22
	1-stearoyl-2-oleoyl-GPG (18:0/18:1)	1.56
	1-stearoyl-GPE (18:0)	1.13
	1-stearoyl-GPS (18:0)	1.22
	1-(1-enyl-palmitoyl)-2-linoleoyl-GPE (P-16:0/18:2)	1.32
	sphingomyelin (d18:2/23:0, d18:1/23:1, d17:1/24:1)	1.33
	N-nervonoyl-sphingadiene (d18:2/24:1)	1.26
	sterol	1.31
	glycosylceramide	1.18
Nucleotides	2'-AMP	0.78
	guanosine-5'-triphosphate	1.60
	5'-GMP	0.86
	uracil	0.85
	nicotinamide riboside	0.77
Co-factors and Vitamins	N1-Methyl-2-pyridone-3-carboxamide	1.57

Asterisk indicated statistical significance with a p-value < 0.05. Fold changes greater than 1 indicate an increase in abundance and fold changes less than one indicate a decrease in metabolite abundance.

### There are sex specific differences in the metabolic profile in response to exposure to prenatal inflammation

**Fig 7:**
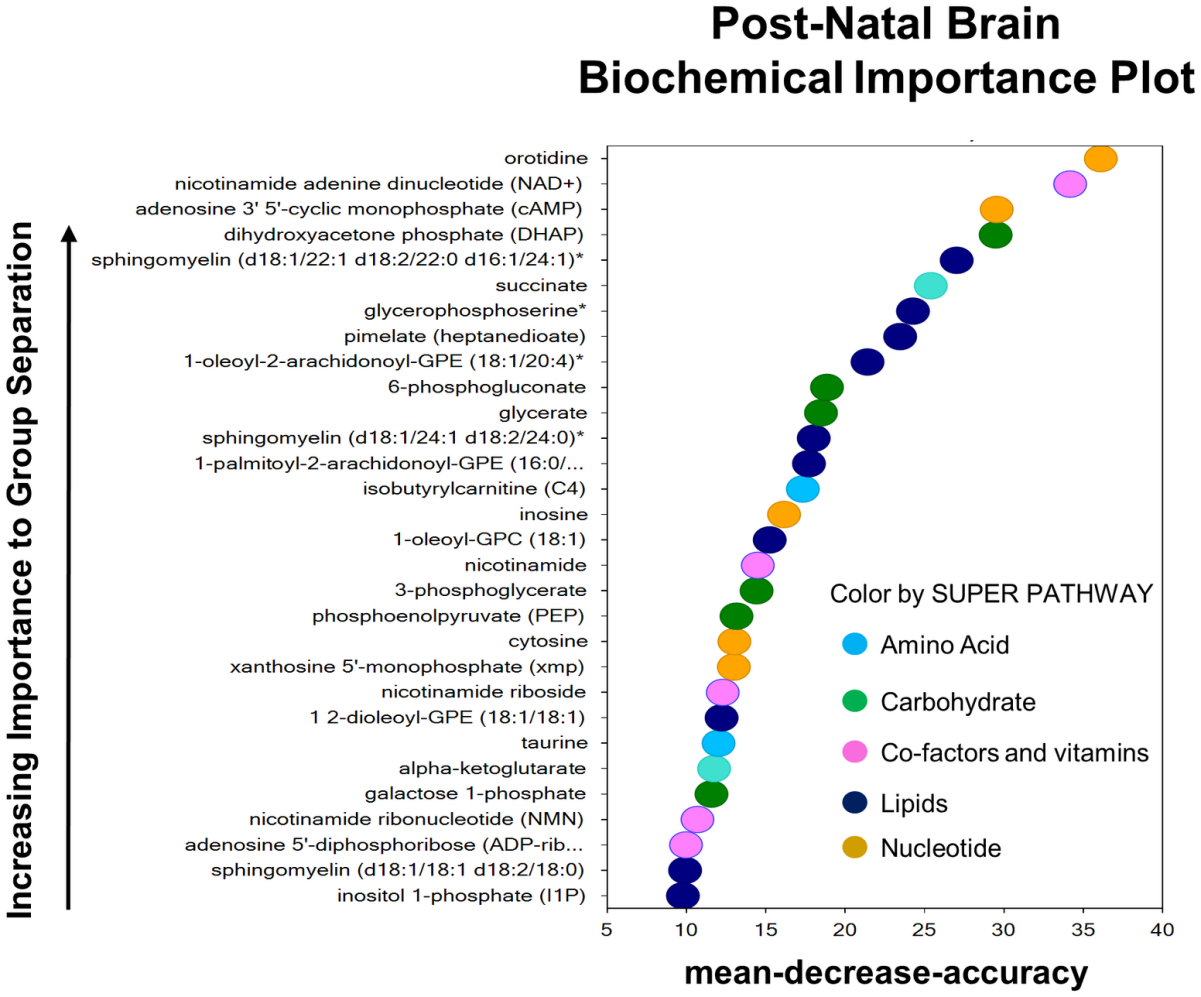
The metabolic profile of the postnatal brain, after prenatal exposure to inflammation. Random forest plot of top 30 biochemicals present in the P1 brain, after prenatal exposure to LPS, based on their importance in separating the metabolic profiles of the saline and LPS exposed groups. The predictive accuracy was 75%.

Exposure to inflammation *in utero* altered the metabolic profile of the postnatal brain. Thirty-eight out of 512 total biochemicals detected were elevated and 13 metabolites had reduced levels (P < 0.05). The 30 biochemicals used to generate the random forest plot, with a predictive accuracy of 75%, belong to the following super pathway families: amino acid, carbohydrate, co-factors and vitamins, lipids, and nucleotides (Fig 7).

#### Lipid metabolism

**Fig 8:**
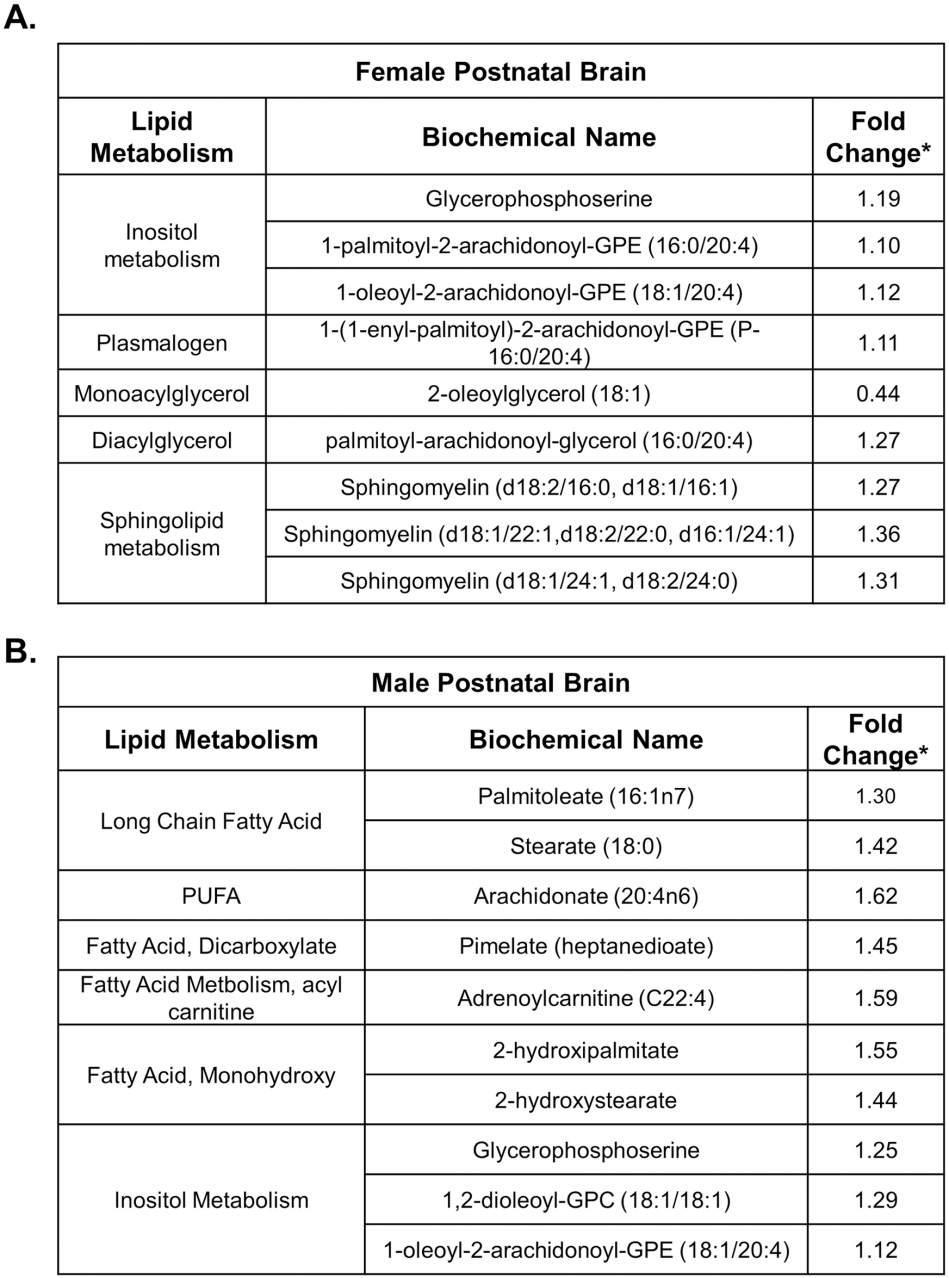
Lipid metabolite profiles of the female and male P2 brains after in utero exposure to inflammation. Statistically significant fold changes (*, p< 0.05) in lipid metabolites found in the female (A) and male (B) brain after prenatal exposure to inflammation.

Male and female brains were analyzed separately and showed statistically significant differences in their lipid compound profiles in response to intrauterine inflammation. Phospho- and lysolipid metabolism was affected in both sexes (S1 Table). However, the alterations in sphingolipids, plasmalogens and diacylglycerols were specific to the female brain, while fatty acids were elevated exclusively in males (Fig 8A and 8B).

#### Energy metabolism

Exposure to inflammation *in utero* resulted in an increased abundance in members of the glycolysis, gluconeogenesis, and pyruvate metabolic pathways in the neonatal brains of both sexes. Upon further investigation, corresponding changes in the males were responsible for the enhancement of DHAP, 3-phosphoglycerate and PEP in the combined cohort (Table 5)

**Table 5:** Alterations in carbohydrate metabolites in the male and female post-natal brain after prenatal exposure to inflammation.

	Biochemical Name	Fold Change*		
		Both sexes	Males	Females
**Glycolysis, gluconeogenesis, and pyruvate metabolism**	isobar	1.58	1.72	1.41
	DHAP	1.74	1.91	1.57
	3-phosphoglycerate	1.41	1.55	1.28
	PEP	1.41	1.59	1.25
	pyruvate	1.27	1.35	1.19

Fold changes greater than 1 indicate a statistically significant (*, p < 0.05) increase in abundance and fold changes less than one indicate a statistically significant (*, p < 0.05) decrease in metabolite abundance.

The aforementioned describes the most significantly altered biochemicals as they pertain to inflammation-induced fetal brain injury. A full list of altered compounds can be found in S1 Table. Statistically significant alterations in metabolite levels have p values < 0.05.

## Discussion

This study reveals the potent effect that intrauterine inflammation has on the metabolic state of the fetus and fetal brain. Our data show that there is an acute response to the inflammatory insult, which resulted in dramatic alterations in the metabolic fingerprint in both the fetal brain and amniotic fluid 6 hours after exposure to inflammation. Of interest for long term neurodevelopment, the metabolic profile remains altered at a later time point but different pathways are affected in contrast to the acute exposure. Most importantly, pups who were exposed to *in utero* inflammation and born at term, demonstrate disruptions in key metabolic profiles that are obligatory to neuronal development and function. Changes in the metabolic profiles are sex-specific, offering a potential explanation as to the sexual dimorphism that exists with certain neurobehavioral conditions.

In the current study, we sought to identify metabolites that may provide insight regarding the mechanisms underlying the pathogenesis of fetal brain injury and/or lead to biomarker discovery. Over the past few decades there has been great interest in discovering novel biomarkers of various perinatal neurological injuries [25,26]. Currently, reliable biomarkers that can accurately predict inflammation-induced fetal brain injury do not exist. Because the amniotic fluid is a reservoir of indicators of fetal health and disease, we probed the metabolome of amniotic fluid collected 6 and 48 hours after exposure to prenatal inflammation. Our study demonstrates a dramatic elevation in purine metabolites, specifically members of the xanthine/hypoxanthine pathway, in the amniotic fluid, 6 hours after insult. It is known that purines are released in the face of inflammation and infection as well as during pathological conditions such as hypoxia/ischemia, stroke or trauma [27–29]. Purines and their metabolites are not only harbingers of inflammation, but are signaling molecules that control neural stem cell function and neurogenesis [30]. Similar to the amniotic fluid at 6 hours, we did detect increases in xanthine/hypoxanthine metabolites in the fetal brain, suggesting that their elevation in the amniotic fluid may be physiologically relevant and associated to changes in fetal brain function [27,30,31]. Others have also shown that elevations in purine metabolites, specifically inosine, may be a defense mechanism against ischemic and traumatic brain injury, suggesting that release of this nucleoside may be protective and a method by which the body is attempting to alleviate the injury [32,33]. Though we cannot fully address the functional relevance of enhanced xanthine/hypoxanthine levels, our results suggest that xanthine/hypoxanthine may be a plausible biomarker of inflammation-induced fetal brain injury. By performing a commercially available assay for xanthine/hypoxanthine, we were able to replicate the metabolomics data for these biochemical compounds, revealing that similar tests can be readily conducted in a clinical setting.

In addition to the previously mentioned compounds, amino acid metabolites were dramatically increased in the amniotic fluid 6 hours after exposure to prenatal inflammation. Specifically, glutamate, histidine, lysine, tryptophan and branched chain amino acid metabolites were elevated in the amniotic fluid. Interestingly, these same families of amino acid metabolites were altered in the fetal brains of our animal model, 6 hours post inflammatory insult suggesting that the metabolic content of the amniotic fluid echoes the fetal metabolome, especially during early/mid gestation [34].

A number of amino acid metabolites that were significantly altered in the fetal brain are known to act as neurotransmitters or their precursors, including GABA, putrescine, aspartate, and glutarate. Enhancement of neurotransmitters or their precursors in the fetal brain will undoubtedly alter synaptic transmission and potentially adversely affect the developing brain. It has been well documented that GABA levels are altered in the brains of patients diagnosed with autism spectrum disorder [35]. Additionally, excessive glutarate levels, found in the CNS of patients deficient in the enzyme responsible for glutarate catabolism, result in severe neurological symptoms [36–38]. Thus, overabundance of neurotransmitters or their metabolites can have grave physiological and psychological consequences for the developing fetal brain.

Similarly, our metabolomic profile demonstrates elevated kynurenate and BCAA levels in the fetal brain 6 hours after exposure to inflammation. Dietary exposure to kynurenate during gestation produces neurochemical and cognitive defects in adulthood that resemble symptoms of schizophrenia [39,40]. Furthermore, investigators have revealed that inflammation stimulates the production of kynurenate and this excess negatively affects glutamatergic and cholinergic signaling in the brain [41,42]. Additionally, *in utero* exposure to increased kynurenine levels resulted in a decrease in dendritic spines, a phenotype that we have shown repeatedly with our model of inflammation-induced brain injury [1,2,39]. Likewise, inflammation-induced enhancement of BCAA and their metabolites adversely affects the function and development of the fetal brain. Specifically, Maple Syrup Urine Disease (MSUD) results from a congenital defect in branched chain ketoacid dehydrogenase, the enzyme responsible for BCAA catabolism [43]. Excessive levels of BCAAs result in neurological and psychiatric deterioration due to their role in neurotransmitter release and replenishment [44]. The inflammation-induced elevated kynurenate and BCAA levels within the context of our model may negatively impact synaptic signaling and neuronal architecture resulting in cognitive and neurobehavioral deficits.

Forty-eight hours post-inflammation there were only 9 significantly altered compounds detected in the amniotic fluid compared to 81 compounds at the 6 hour time point. The most striking finding at the 48 hour time-point was the 15-fold (p<0.05) enhancement of 4-ethylphenylsulfate (4-EPS). Interestingly, this compound is a bioactive, benzoate metabolite, which was linked to anxiety-like behavioral abnormalities in offspring from a mouse model of viral infection [45]. Similar to our findings with xanthine/hypoxanthine, 4-EPS may be a useful biomarker of inflammation-induced neuronal injury and warrants further investigation to better understand its physiological effects and potential clinical benefits.

Within the fetal brain, we detected a significant decline in lipid metabolites, specifically lysolipids, sphingolipids, monoacylglycerols and sterols at the 48 hour time point. Sphingolipids, the derivatives of sphingomyelin, play an active role in innate and adaptive immunity [46]. Both sphinganine and N-stearoyl-sphingosine are significantly decreased suggesting a disturbance in the sphingomyelin signaling pathway. Additionally, at 48 hours, cholesterol and desmosterol are both significantly decreased in the fetal brain. Cholesterol is a key myelin component imperative for synaptogenesis and neurotransmitter release [47] and desmosterol is the penultimate precursor to cholesterol, which accumulates rapidly in the murine brain between E14 and P10 [48]. Both of these metabolites are required in such high volumes during brain development that even minor declines in their bioavailability may negatively impact synaptic transmission and disrupt normal brain function.

While this study provides insight into the fetal metabolome, it also demonstrates that at baseline, male and female brains have unique metabolic profiles and an *in utero* insult alters brain metabolites in a sex specific manner. Several neurological and psychiatric illnesses have pronounced sex differences in presentation. Dyslexia, autism, ADHD and Tourette’s syndrome are all more prevalent in men, while Rett syndrome, dementia and PTSD are more common in women [11]. Our data demonstrate that post-exposure to inflammation, the brains of males showed an increase in fatty acid metabolites, while female brains had elevated sphingolipids, suggesting that inflammation uniquely affects the lipid profile in the male and female brain. The alterations in the lipid profiles in male and females neonates is a critical finding in that lipids make up more than 50% of the dry weight of the brain, are the major component of the cell membranes, are repositories of chemical energy and play key role in signal transduction and cell signaling [49,50]. Disruption of lipid metabolism in the neonate is a consequence of prenatal exposure to inflammation is sex-specific and may be a key event in the pathogenesis of inflammation-induced brain injury.

The alteration in the lipid patterns was not the only difference between the males and female neonates, both sexes had increases in metabolites derived from the glycolysis, gluconeogenesis and pyruvate metabolism. While both sexes demonstrated an increase, the males’ neonatal brain had a greater abundance of the energy-related biochemicals, suggesting that the sexual dimorphism in inflammation-induced changes in the metabolome affect multiple pathways of the neonatal brain. Taken together, we conclude that the sex of the offspring will impact the neurological impairment that results from exposure to prenatal inflammation.

In addition to exploring the effect sex has on the response to inflammation, this study specifically probes brain tissue and provides a metabolomic snapshot over time of fetal and neonatal brain physiology after exposure to prenatal inflammation. While we cannot dissect apart which components of the brain, neurons or glia, are most dramatically affected by the inflammatory insult, we can begin to explore some of the relevant pathways that play a role in brain physiology at large. Few researchers have employed metabolomics to profile brain tissue collected after injury. Jaeger and coworkers demonstrated that the different regions of the mouse brain have unique metabolomic signatures and that excitotoxic injury results in an altered brain metabolome [51]. Similarly, utilizing an excitotoxic lesion model, Blaise and coworkers demonstrated an altered metabolomic fingerprint of the neonatal brain, post injury [52]. It is clear that defining the metabolomic profile of affected tissue is informative and may provide insight into the pathophysiology of brain injury.

In conclusion, these studies demonstrate that exposure to prenatal inflammation drastically alters the metabolomic profile of the amniotic fluid and fetal brain. These metabolomics changes persist into postnatal life, are sex-specific and may explain the sexual diversity in neurobehavioral phenotypes associated with exposure to prenatal inflammation.

## Supporting information

S1 Table Metabolic compounds, with altered levels in response to intrauterine inflammation in the amniotic fluid and the fetal and postnatal brain (P < 0.05). (DOCX)
