# Supplementary material for: Exposure to intrauterine inflammation alters metabolomic profiles in the amniotic fluid, fetal and neonatal brain in the mouse
Source: PLoS One. 2017 Oct 19;12(10):e0186656. doi: 10.1371/journal.pone.0186656 (PMC5648237; doi:10.1371/journal.pone.0186656)
Supplement: S1 Table — (DOCX) [file pone.0186656.s001.docx]

| **Pathway** | **Biochemical Name** | ***6h*** | | ***48h*** | | ***P1*** | | | |
| --- | --- | --- | --- | --- | --- | --- | --- | --- | --- |
|  |  | ***FB*** | ***AF*** | ***FB*** | ***AF*** | ***All*** | ***F*** | | ***M*** |
| Amino Acids and Peptides | *Glycine, Serine and Threonine Metabolism* | | | | | | | | |
|  | N-acetylglycine | 1.2 |  | 1.3 |  |  | |  |  |
|  | N-acetylserine | 1.2 |  |  |  |  | |  |  |
|  | *Alanine and Aspartate Metabolism* | | | | | | | | |
|  | aspartate | 0.6 |  | 0.9 |  |  | |  |  |
|  | *Glutamate Metabolism* | | | | | | | | |
|  | glutamate | 0.9 |  |  |  |  | |  |  |
|  | γ-aminobutyrate | 1.3 | 3.5 |  |  |  | |  |  |
|  | glutamate, γ-methyl ester | 0.5 |  |  |  |  | |  |  |
|  | *Histidine Metabolism* | | | | | | | | |
|  | histidine | 1.4 |  |  |  |  | |  |  |
|  | N-acetylhistidine | 1.4 | 1.4 | 1.9 |  |  | |  |  |
|  | 1-methylhistamine | 1.8 |  |  |  |  | |  |  |
|  | imidazole propionate |  |  |  |  | 1.5 | |  |  |
|  | thioproline |  |  |  |  | 1.3 | |  |  |
|  | 3-methylhistidine |  | 1.5 |  |  |  | |  |  |
|  | *Lysine Metabolism* | | | | | | | | |
|  | lysine | 1.4 |  |  |  |  | |  |  |
|  | N2-acetyllysine |  | 2 |  |  |  | |  |  |
|  | glutarate | 1.3 | 3.4 |  |  |  | |  |  |
| Amino Acids and Peptides | N2,N6-diacetyllysine |  | 1.3 |  |  |  | |  |  |
|  | *Phenylalanine and Tyrosine Metabolism* | | | | | | | | |
|  | 3-(4-hydroxyphenyl)  lactate | 1.5 |  |  |  |  | |  |  |
|  | *Tryptophan Metabolism* | | | | | | | | |
|  | tryptophan | 1.5 |  |  |  |  | |  |  |
|  | indolelactate | 2.3 |  |  |  |  | |  |  |
|  | 3-indoxyl sulfate | 1.8 |  |  |  |  | |  |  |
|  | kynurenine |  |  | 1.4 |  |  | |  |  |
|  | kynurenate | 2.2 | 3 |  |  |  | |  |  |
|  | anthranilate |  | 2 |  |  |  | |  |  |
|  | indole-3-carboxylic acid |  | 1.4 |  |  |  | |  |  |
|  | *Leucine, Isoleucine and Valine Metabolism* | | | | | | | | |
|  | leucine | 1.4 |  |  |  |  | |  |  |
|  | N-acetylleucine | 2.1 | 2.9 |  |  |  | |  |  |
|  | 4-methyl-2-oxopentanoate | 9.4 | 1.3 |  |  |  | |  |  |
|  | β-hydroxyisovaleroyl  carnitine | 0.9 | 1.4 |  |  |  | |  |  |
|  | α-hydroxyisovalerate | 1.9 | 1.5 |  |  |  | |  |  |
|  | methylsuccinate |  | 1.3 | 1.6 |  |  | |  |  |
|  | isoleucine | 1.4 | 1.3 |  |  |  | |  |  |
|  | N-acetylisoleucine | 1.5 | 0.8 |  |  |  | |  |  |
|  | 3-methyl-2-oxovalerate | 8 |  |  |  |  | |  |  |
|  | 2-methylbutyrylcarnitine | 1.7 | 1.4 |  |  |  | |  |  |
|  | 2-hydroxy-3-methylvalerate | 2.5 |  |  |  |  | |  |  |
|  | ethylmalonate | 1.2 |  |  |  |  | |  |  |
|  | valine | 1.6 |  |  |  |  | |  |  |
|  | 3-hydroxyisobutyrate | 0.8 |  |  |  |  | |  |  |
|  | α-hydroxyisocaproate | 2.9 |  |  |  |  | |  |  |
|  | *Methionine, Cysteine, SAM and Taurine Metabolism* | | | | | | | | |
|  | N-formylmethionine | 0.8 |  |  |  |  | |  |  |
|  | cystathionine |  |  | 0.8 |  |  | |  |  |
|  | cystine |  |  |  | 0.6 |  | |  |  |
|  | cysteine | 1.6 |  |  |  |  | |  |  |
|  | S-methylcysteine |  |  |  |  |  | |  | 0.7 |
|  | cysteine sulfinic acid |  | 2.8 |  |  |  | |  |  |
|  | hypotaurine |  |  | 0.8 |  |  | |  |  |
|  | taurine |  |  |  |  | 0.9 | |  | 0.9 |
|  | *Urea cycle; Arginine and Proline Metabolism* | | | | | | | | |
|  | urea | 1.8 | 1.3 |  |  |  | |  |  |
|  | ornithine |  |  | 0.8 |  |  | |  |  |
|  | proline | 1.4 |  |  |  |  | |  |  |
|  | argininosuccinate |  |  |  |  | 1.3 | | 1.4 |  |
|  | argininate |  |  |  |  | 0.9 | |  | 0.8 |
| Amino Acids and Peptides | N-methylproline |  | 1.1 |  |  |  | |  |  |
|  | *Creatine Metabolism* | | | | | | | | |
|  | creatine | 0.9 |  |  |  |  | |  |  |
|  | creatinine |  |  | 1.1 |  |  | |  |  |
|  | *Polyamine Metabolism* | | | | | | | | |
|  | putrescine | 1.1 |  |  |  |  | |  |  |
|  | 4-acetamidobutanoate |  |  | 2.1 |  |  | |  |  |
|  | *Guanidino and Acetamido Metabolism* | | | | | | | | |
|  | 1-methylguanidine | 0.8 |  |  |  |  | |  |  |
|  | 4-guanidinobutanoate |  | 2 |  |  |  | |  |  |
|  | *Glutathione Metabolism* | | | | | | | | |
|  | cysteine-glutathione disulfide |  |  | 1.9 |  |  | |  |  |
|  | S-methylglutathione | 0.9 |  |  |  |  | |  |  |
|  | S-lactoylglutathione | 0.5 |  |  |  |  | |  |  |
|  | ophthalmate | 0.6 |  |  |  |  | |  |  |
|  | *Dipeptide Derivative* | | | | | | | | |
|  | anserine |  |  |  |  | 1.5 | |  |  |
|  | *Dipeptides* | | | | | | | | |
|  | phenylalanylglycine |  |  |  | 0.7 |  | |  |  |
|  | *Gamma-glutamyl Amino Acid* | | | | | | | | |
|  | γ-glutamylthreonine | 0.7 |  |  |  |  | |  |  |
|  | γ-glutamyltryptophan | 1.8 |  |  |  |  | |  |  |
| Carbohydrates | *Glycolysis, Gluconeogenesis, and Pyruvate Metabolism* | | | | | | | | |
|  | pyruvate |  | 0.8 |  |  | 1.3 | |  |  |
|  | glycerate |  |  |  |  | 0.9 | | 0.9 |  |
|  | 3-phosphoglycerate |  |  |  |  | 1.4 | |  | 1.6 |
|  | 1,5-anhydroglucitol |  | 0.9 |  |  |  | |  |  |
|  | glucose |  | 0.2 |  |  |  | |  |  |
|  | fructose-6-phosphate | 0.5 |  |  |  |  | |  |  |
|  | Isobar: fructose 1,6-diphosphate* | 0.3 |  |  |  | 1.6 | |  |  |
|  | dihydroxyacetone phosphate | 0.5 |  |  |  | 1.7 | | 1.6 | 1.9 |
|  | phosphoenolpyruvate |  |  |  |  | 1.4 | |  | 1.6 |
|  | lactate | 1.3 | 1.2 |  |  |  | |  |  |
|  | *Pentose Phosphate Pathway* | | | | | | | | |
|  | arabonate/xylonate |  | 0.8 |  |  |  | |  |  |
|  | sedoheptulose-7-phosphate | 0.6 |  |  |  | 1.2 | |  |  |
|  | ribulose/xylulose 5-phosphate | 0.6 |  |  |  |  | |  |  |
|  | 6-phosphogluconate |  |  |  |  | 4.5 | |  | 5.8 |
|  | *Pentose Metabolism* | | | | | | | | |
|  | ribonate |  |  | 1.2 |  |  | |  |  |
|  | arabinose |  | 0.7 |  |  |  | |  |  |
|  | *Glycogen Metabolism* | | | | | | | | |
|  | maltotetraose |  | 0.3 |  |  |  | |  |  |
|  | maltotriose |  | 0.3 |  |  |  | |  |  |
| Carbohydrates | maltose | 0.6 | 0.5 |  |  |  | |  |  |
|  | *Fructose, Mannose and Galactose Metabolism* | | | | | | | | |
|  | fructose | 0.6 | 0.2 |  |  |  | |  |  |
|  | mannitol/sorbitol | 0.8 |  | 1.1 |  |  | |  |  |
|  | galactose 1-phosphate |  |  |  |  | 1.5 | |  |  |
|  | *Nucleotide Sugar* | | | | | | | | |
|  | UDP-glucose | 0.5 |  |  |  |  | |  |  |
|  | UDP-galactose | 0.5 |  |  |  |  | |  |  |
|  | UDP-glucuronate | 0.6 |  |  |  |  | |  |  |
|  | cytidine 5'-monophospho-N-acetylneuraminic acid | 0.7 |  |  |  |  | |  |  |
|  | *Advanced Glycation End-product Metabolism* | | | | | | | | |
|  | N6-carboxymethyllysine |  | 1.3 |  |  |  | |  |  |
|  | *Aminosugar Metabolism* | | | | | | | | |
|  | glucuronate |  | 0.8 |  |  |  | |  |  |
|  | *Long Chain Fatty Acid* | | | | | | | | |
|  | palmitate (16:0) | 1.3 |  |  |  |  | |  |  |
|  | palmitoleate (16:1n7) | 1.7 |  |  |  |  | |  | 1.3 |
|  | stearate (18:0) |  |  |  |  |  | |  | 1.4 |
|  | oleate/vaccenate (18:1) | 1.6 |  |  |  |  | |  |  |
|  | *Polyunsaturated Fatty Acid (n3 and n6)* | | | | | | | | |
|  | eicosapentaenoate | 1.3 | 2.5 |  | 1.3 |  | |  |  |
|  | docosahexaenoate | 1.2 | 1.5 |  |  |  | |  |  |
|  | linoleate (18:2n6) | 1.5 |  |  |  |  | |  |  |
|  | linolenate | 1.3 |  |  | 1.3 |  | |  |  |
|  | dihomo-linolenate | 1.5 |  |  |  |  | |  |  |
|  | arachidonate | 1.6 |  |  |  | 1.4 | |  | 1.6 |
|  | docosapentaenoate | 1.2 |  |  |  |  | |  |  |
|  | dihomo-linoleate | 1.3 |  |  |  |  | |  |  |
|  | *Fatty Acid Metabolism (also BCAA Metabolism)* | | | | | | | | |
|  | butyrylcarnitine | 0.7 |  |  |  |  | |  |  |
|  | methylmalonate | 1.7 |  |  |  |  | |  |  |
|  | pimelate |  |  |  |  | 1.3 | |  | 1.5 |
|  | *Fatty Acid Metabolism(Acyl Carnitine)* | | | | | | | | |
|  | decanoylcarnitine |  | 3.1 |  |  |  | |  |  |
|  | myristoylcarnitine |  | 4 |  |  |  | |  |  |
|  | palmitoylcarnitine |  | 3.8 |  |  |  | |  |  |
|  | stearoylcarnitine |  | 2.5 |  |  |  | |  |  |
|  | linoleoylcarnitine |  | 5 |  |  |  | |  |  |
|  | oleoylcarnitine |  | 4.2 |  |  |  | |  |  |
|  | myristoleoylcarnitine |  | 3.1 |  |  |  | |  |  |
|  | adrenoylcarnitine |  |  |  |  |  | |  | 1.6 |
|  | *Carnitine Metabolism* | | | | | | | | |
| Lipids | carnitine | 0.8 |  |  |  |  | |  |  |
|  | *Fatty Acid, Monohydroxy* | | | | | | | | |
|  | 3-hydroxyoctanoate |  | 3.4 |  |  |  | |  |  |
|  | 13-HODE + 9-HODE | 1.5 |  |  |  |  | |  |  |
|  | 2-hydroxypalmitate |  |  |  |  |  | |  | 1.6 |
|  | 2-hydroxystearate |  |  |  |  |  | |  | 1.4 |
|  | *Eicosanoid* | | | | | | | | |
|  | 5-HETE | 3.6 |  |  |  |  | |  |  |
|  | *Endocannabinoid* | | | | | | | | |
|  | palmitoyl ethanolamide | 6.3 |  |  |  |  | |  |  |
|  | *Inositol Metabolism* | | | | | | | | |
|  | inositol 1-phosphate |  |  |  |  | 1.2 | |  |  |
|  | *Phospholipid/lysolipid/Lysoplasmalogen Metabolism* | | | | | | | | |
|  | glycerophosphoserine |  |  |  |  | 1.2 | | 1.2 | 1.3 |
|  | 1,2-dioleoyl-GPC |  |  |  |  |  | |  | 1.3 |
|  | 1-palmitoyl-2-arachidonoyl-GPE (16:0/20:4) |  |  |  |  | 1.1 | | 1.1 |  |
|  | 1,2-dioleoyl-GPE (18:1/18:1) |  |  |  |  | 1.2 | |  |  |
|  | 1-oleoyl-2-arachidonoyl-GPE (18:1/20:4) |  |  |  |  | 1.1 | | 1.1 | 1.1 |
|  | choline | 1.2 | 2.5 |  |  |  | |  |  |
|  | glycerophosphorylcholine | 0.4 |  |  |  |  | |  |  |
|  | ethanolamine | 3.4 |  |  |  |  | |  |  |
| Lipids | cytidine-5'-diphosphoethanolamine | 0.7 |  |  |  |  | |  |  |
|  | glycerophosphoethanolamine | 0.6 |  |  |  |  | |  |  |
|  | trimethylamine N-oxide |  | 1.1 |  |  |  | |  |  |
|  | *Lisolipid* | | | | | | | | |
|  | 1-palmitoyl-2-arachidonoyl-GPI (16:0/20:4) |  |  | 0.9 |  |  | |  |  |
|  | 1-palmitoyl-2-oleoyl-GPG (16:0/18:1) |  |  | 0.9 |  |  | |  |  |
|  | 1-palmitoyl-2-arachidonoyl-GPE (16:0/20:4) |  |  | 0.9 |  |  | |  |  |
|  | 1-palmitoleoyl-2-linoleoyl-GPC (16:1/18:2) | 0.8 |  |  |  | 1.2 | |  |  |
|  | 1-oleoyl-2-arachidonoyl-GPI (18:1/20:4) |  |  | 0.9 |  |  | |  |  |
|  | 2-palmitoyl-GPC (16:0) |  | 0.8 |  |  |  | |  |  |
|  | *Plasmalogen* | | | | | | | | |
|  | 1-(1-enyl-palmitoyl)-2-arachidonoyl-GPE (P-16:0/20:4) |  |  |  |  | 1.1 | | 1.1 |  |
|  | 1-(1-enyl-stearoyl)-2-oleoyl-GPE (P-18:0/18:1) |  |  |  |  | 1.1 | |  |  |
|  | 1-(1-enyl-stearoyl)-2-arachidonoyl-GPE (P-18:0/20:4) |  |  |  |  | 1.1 | |  |  |
|  | 1-palmitoleoyl-GPC (16:1) |  | 0.8 |  |  |  | |  |  |
|  | 1-arachidonoyl-GPC (20:4) |  | 0.7 |  |  |  | |  |  |
| Lipids | 1-linoleoyl-GPA (18:2) |  | 0.5 |  |  |  | |  |  |
|  | *Glycerolipid Metabolism* | | | | | | | | |
|  | glycerol | 1.9 | 1.6 |  |  |  | |  |  |
|  | glycerophosphoglycerol | 0.8 |  |  |  |  | |  |  |
|  | glycerol 3-phosphate |  |  |  |  | 0.8 | |  |  |
|  | *Monoacylglycerol* | | | | | | | | |
|  | palmitoyl-docosahexaenoyl-glycerol (16:0/22:6) |  |  | 0.8 |  |  | |  |  |
|  | palmitoyl-docosahexaenoyl-glycerol (16:0/22:6) |  |  | 0.8 |  |  | |  |  |
|  | stearoyl-docosahexaenoyl-glycerol (18:0/22:6) |  |  | 0.8 |  |  | |  |  |
|  | 2-oleoylglycerol (18:1) |  |  |  |  |  | | 0.4 |  |
|  | *Diacylglycerol* | | | | | | | | |
|  | oleoyl-linoleoyl-glycerol (18:1/18:2) |  |  |  |  | 1.2 | |  |  |
|  | palmitoyl-arachidonoyl-glycerol (16:0/20:4) |  |  |  |  | 1.2 | | 1.3 |  |
|  | palmitoyl-oleoyl-glycerol (16:0/18:1) |  |  |  |  | 1.2 | |  |  |
|  | stearoyl-arachidonoyl-glycerol (18:0/20:4) |  |  |  |  | 1.2 | |  |  |
|  | *Sphingolipid Metabolism* | | | | | | | | |
|  | sphinganine | 0.5 |  | 0.9 |  |  | |  |  |
|  | sphingosine | 0.7 |  |  |  |  | |  |  |
| Lipids | sphingosine 1-phosphate |  | 0.6 |  | 0.7 |  | |  |  |
|  | N-stearoyl-sphingosine (d18:1/18:0) |  |  | 0.8 |  |  | |  |  |
|  | sphingomyelin (d18:2/16:0, d18:1/16:1) |  |  |  |  | 1.2 | | 1.3 |  |
|  | sphingomyelin (d18:1/18:1, d18:2/18:0) |  |  |  |  | 1.2 | |  |  |
|  | sphingomyelin (d18:1/22:1, d18:2/22:0, d16:1/24:1) |  |  |  |  | 1.2 | | 1.4 |  |
|  | sphingomyelin (d18:1/24:1, d18:2/24:0) |  |  |  |  | 1.2 | | 1.3 |  |
|  | sphingosine |  |  |  |  | 1.1 | |  |  |
|  | sphingomyelin (d18:0/20:0, d16:0/22:0) |  |  |  |  | 1.2 | |  |  |
|  | *Sterol* | | | | | | | | |
|  | desmosterol | 0.9 |  | 0.9 |  |  | |  |  |
|  | cholesterol | 0.8 |  | 0.9 |  |  | |  |  |
|  | *Steroid* | | | | | | | | |
|  | corticosterone |  | 4 |  |  |  | |  |  |
| Nucleotides | *Purine Metabolism, (Hypo)Xanthine/Inosine containing* | | | | | | | | |
|  | inosine |  | 3.9 | 0.9 |  | 1.1 | | 1.1 |  |
|  | hypoxanthine | 1.7 | 23 |  |  |  | |  |  |
|  | xanthine | 2.4 | 9.9 |  |  |  | |  |  |
|  | xanthosine | 8.9 |  |  |  |  | |  |  |
|  | xanthosine 5'-monophosphate |  |  |  |  | 0.8 | | 0.8 |  |
|  | urate | 2.3 | 2.8 |  |  |  | |  |  |
|  | methylphosphate |  |  |  |  | 1.1 | | 1.2 |  |
|  | *Purine Metabolism, Adenine containing* | | | | | | | | |
|  | adenosine 5'-monophosphate | 0.7 |  |  |  |  | |  |  |
|  | adenosine 3'-monophosphate | 0.5 |  |  |  |  | |  |  |
|  | adenosine 2'-monophosphate | 0.5 |  |  |  |  | |  | 0.7 |
|  | adenylosuccinate | 0.5 |  |  |  |  | |  |  |
|  | N6-succinyladenosine |  | 2.1 |  |  |  | |  |  |
|  | adenosine 3',5'-cyclic monophosphate |  |  |  |  | 1.4 | | 1.4 | 1.4 |
|  | N1-methyladenosine |  | 2 |  |  |  | |  |  |
|  | *Purine Metabolism, Guanine containing* | | | | | | | | |
|  | guanosine 5'- monophosphate | 0.9 |  |  |  |  | |  |  |
|  | guanosine |  | 5.8 |  |  |  | |  |  |
|  | guanine |  | 11 |  |  |  | |  |  |
|  | N2,N2-dimethylguanosine | 3.3 | 1.7 |  |  |  | |  |  |
|  | *Pyrimidine Metabolism, Orotate containing* | | | | | | | | |
|  | dihydroorotate |  |  |  |  |  | |  | 0.6 |
|  | orotidine | 0.9 |  |  |  | 0.9 | |  | 0.9 |
|  | *Pyrimidine Metabolism, Uracil containing* | | | | | | | | |
|  | uridine 5'-monophosphate | 0.6 |  | 1.3 |  |  | |  |  |
|  | uridine |  | 3 | 0.9 |  |  | |  |  |
|  | uracil | 2.2 | 15 |  |  |  | |  |  |
|  | pseudouridine | 1.2 |  |  |  |  | |  |  |
|  | 5-methyluridine | 3.6 |  |  |  |  | |  |  |
|  | β-alanine | 0.8 |  |  |  |  | |  |  |
|  | N-acetyl-β-alanine |  |  | 1.6 |  |  | |  |  |
|  | 5,6-dihydrouracil |  | 2.2 |  |  |  | |  |  |
|  | *Pyrimidine Metabolism, Cytidine containing* | | | | | | | | |
|  | cytidine 5'-monophosphate | 0.6 |  |  |  |  | |  |  |
|  | cytidine 2',3'-cyclic monophosphate |  |  | 0.9 |  |  | |  |  |
|  | 2'-deoxycytidine 5'-monophosphate | 0.6 |  |  |  |  | |  |  |
|  | *Pyrimidine Metabolism, Cytidine containing* | | | | | | | | |
|  | 5-methyl-2'-deoxycytidine | 0.7 | 0.5 | 0.7 |  |  | |  |  |
|  | *Pyrimidine Metabolism, Thymine containing* | | | | | | | | |
|  | thymine | 3.5 | 17 |  |  |  | |  |  |
|  | 5,6-dihydrothymine |  | 2.7 |  |  |  | |  |  |
| Energy Metabolism | *TCA Cycle* | | | | | | | | |
|  | α-ketoglutarate | 1.1 |  |  |  | 0.8 | |  |  |
|  | succinate |  | 3.1 |  |  | 0.6 | | 0.6 | 0.6 |
|  | fumarate |  | 1.5 |  |  |  | |  |  |
|  | malate | 1 | 1.9 |  |  | 1.1 | | 1.2 |  |
|  | *Oxidative Phosphorylation* | | | | | | | | |
|  | acetylphosphate |  |  |  |  |  | |  | 1.2 |
|  | phosphate | 1.1 |  |  |  |  | |  |  |
|  | *Oxidative Phosphorylation* | | | | | | | | |
|  | acetylphosphate |  |  |  |  |  | |  | 1.2 |
|  | phosphate | 1.1 |  |  |  |  | |  |  |
|  | *Nicotinate and Nicotinamide Metabolism* | | | | | | | | |
| Vitamins, cofactors, xenobotics | nicotinamide |  | 3 |  | 1.5 | 1.1 | | 1.1 |  |
|  | nicotinamide ribonucleotide | 1.7 |  |  |  |  | | 0.8 |  |
|  | nicotinamide N-oxide |  | 1.7 |  |  |  | |  |  |
|  | N1-Methyl-2-pyridone-5-carboxamide | 2 |  |  |  |  | |  |  |
|  | nicotinamide riboside |  |  |  |  | 0.8 | |  | 0.7 |
|  | nicotinamide adenine dinucleotide |  |  |  |  | 0.6 | | 0.5 | 0.7 |
|  | adenosine 5'-diphosphoribose |  |  |  |  | 0.6 | | 0.3 |  |
|  | N1-Methyl-2-pyridone-5-carboxamide |  | 1.4 |  |  |  | |  |  |
|  | *Ascorbate and Aldarate Metabolism* | | | | | | | | |
|  | ascorbate (Vitamin C) |  |  | 0.7 |  |  | |  |  |
|  | threonate |  | 0.8 |  |  | 0.9 | | 0.8 |  |
|  | oxalate (ethanedioate) |  |  |  |  | 0.9 | |  |  |
|  | *Folate Metabolism* | | | | | | | | |
|  | 5-methyltetrahydrofolate | 0.7 |  |  |  |  | |  |  |
|  | *Food Component/Plant* | | | | | | | | |
|  | homostachydrine | 1.3 |  |  |  |  | |  |  |
|  | stachydrine | 1.3 |  |  |  |  | |  |  |
|  | *Bacterial/Fungal* | | | | | | | | |
|  | tartronate (hydroxymalonate) | 0.9 |  |  |  |  | |  |  |
|  | 6-oxopiperidine-2-carboxylic acid | 0.7 |  |  |  |  | |  |  |
|  | palmitoylcholine | 2.2 | 4.2 |  |  |  | |  |  |
|  | *Vitamin B6 Metabolism* | | | | | | | | |
|  | pyridoxal |  | 0.7 |  |  |  | | 1.9 |  |
|  | *Benzoate Metabolism* | | | | | | | | |
|  | 4-ethylphenylsulfate |  |  |  | 16 |  | |  |  |
|  | gluconate |  | 0.7 |  |  |  | |  |  |
|  | daidzein |  | 0.5 |  |  |  | |  |  |
|  | homostachydrine |  | 1.3 |  |  |  | |  |  |
|  | N-glycolylneuraminate |  | 1.6 |  |  |  | |  |  |
|  | stachydrine |  | 1.2 |  |  |  | |  |  |
|  | *Hemoglobin and Porphyrin Metabolism* | | | | | | | | |
|  | heme |  |  |  | 3.7 |  | |  |  |
|  | bilirubin |  |  |  | 0.7 |  | |  |  |

* Isobar: fructose 1,6-diphosphate* glucose 1,6-diphosphate, myo-inositol 1,4 or 1,3-diphosphate
